# Emerging adult perceptions of higher-risk cannabis consumption behaviours

**DOI:** 10.1186/s12954-023-00860-4

**Published:** 2023-09-07

**Authors:** Isobel McMahon, Laura M. Harris-Lane, Jennifer Donnan, Lisa Bishop, Nick Harris

**Affiliations:** 1 Department of Psychology, Memorial University of Newfoundland, St. John’s, NL Canada; 2 School of Pharmacy, Memorial University of Newfoundland, St. John’s, NL Canada; 3 Faculty of Medicine, Memorial University of Newfoundland, St. John’s, NL Canada

**Keywords:** Cannabis, Emerging adults, Perceived risk, Lower-Risk Cannabis Use Guidelines, Harm reduction, Cannabis health literacy

## Abstract

**Background:**

Emerging adults have the highest cannabis consumption rates in Canada and are among the most vulnerable to cannabis-related harms. Since certain cannabis consumption behaviours carry greater risks of harm, the Lower-Risk Cannabis Use Guidelines (LRCUG) provide harm reduction strategies. To address a critical gap in the literature, the current study examined emerging adults’ awareness of the guidelines and perceptions of higher-risk cannabis consumption behaviours identified within the LRCUG.

**Methods:**

Emerging adults (*N* = 653) between the ages of 18–25 years were recruited from across Canada. Participants were presented with five vignettes depicting a character’s cannabis consumption behaviours. Each vignette focused on a unique aspect of the character’s consumption (frequency, polysubstance use, family history of mental illness, method of consumption, and potency). Participants were randomly assigned to one of three conditions within each of the five vignettes that were altered to capture varying levels of risk (e.g. weekly, almost daily, or daily consumption). Following each vignette, participants were asked to respond to four items relating to overall risk of harm, cognitive health, physical health, and mental health.

**Results:**

Participants perceived: (1) frequent consumption to be associated with greater risks than less frequent consumption; (2) simultaneous consumption of cannabis and tobacco as being associated with higher risk of harm, yet no difference between simultaneous consumption of cannabis and alcohol or cannabis consumption alone; (3) consuming cannabis with a family history of psychosis or substance use disorder as being associated with greater overall risk than consumption with no family history; (4) smoking and vaping cannabis as associated with more risk than ingesting edibles; and (5) higher-potency THC-dominant strains as being associated with more risk than lower-potency CBD-dominant strains, yet no difference between the two higher-potency THC-dominant strains.

**Conclusions:**

While emerging adults seemed to appreciate the risks associated with some cannabis consumption behaviours, they had difficulty identifying appropriate levels of harm of other higher-risk behaviours. Through an improved understanding of emerging adult perceptions, effective education campaigns should be designed to improve the awareness of cannabis risks and encourage the uptake of harm reduction awareness and strategies.

**Supplementary Information:**

The online version contains supplementary material available at 10.1186/s12954-023-00860-4.

## Introduction

Since non-medical cannabis was legalized in 2018, Canadian emerging adults between the ages of 20–24 have continued to report the highest rates of past-year (50%) and daily or almost daily cannabis consumption (37%), with an average age of first-time consumption at 20.5 years [1]. Emerging adults are also among the most vulnerable to potential cannabis-related harms across health domains (e.g. cognitive functioning, physical health, and mental health) [2]. Despite the high rates of consumption and risk of harm related to emerging adult cannabis consumption, little research has explored this age group’s perceptions of higher-risk cannabis consumption behaviours. Further, the existing literature on emerging adult perceptions of cannabis consumption suggests that they may not appreciate some of the increased risks associated with cannabis consumption during this developmental phase [3–5].

Emerging adults who consume cannabis daily or almost daily are at an increased risk of experiencing harm to their cognitive health, brain development, physical health, and mental health. Specifically, cannabis consumption in emerging adulthood (up to 25 years of age) has potential harms for the developing brain [6] and has been associated with cognitive impairments related to learning, executive functioning, processing speed, and working memory [7]. Some research suggests that cognitive impairment may be greatly decreased after 72 h of abstinence [7], suggesting that individuals who consume cannabis daily or almost daily may not consistently experience improved cognitive performance. Moreover, cannabis consumers are at an increased risk of stroke and transient ischaemic attack, lower bone density leading to high bone turnover and increased risk of fractures, and negative impacts on pulmonary health (e.g. increased rates of cough, wheezing, and shortness of breath) [8–10]. Given that prolonged exposure to cannabis is associated with negative impacts on pulmonary health, it is possible that young adults lack the foresight into their future health to help inform their more immediate decisions to consume cannabis. Cannabis consumption may also be associated with an increased risk of harm to emerging adults’ mental health. For instance, early initiation of cannabis consumption (i.e. before 25 years of age), frequent consumption, and consuming cannabis with a high THC content have been associated with an increased risk of psychosis, although genetic and environmental factors also influence this relationship [11–15]. Consuming high-potency cannabis has also been associated with exacerbated cannabis use disorder (CUD) symptoms and increased demand for treatment [16, 17]. Further, cannabis consumption, particularly frequent consumption and/or onset of consumption in adolescence, may also be associated with anxiety disorders and major depressive disorder [2, 12].

Given that certain cannabis consumption behaviours can be associated with increased risks of harm, it is important for the general public to have access to reliable and evidence-based information, so individuals can make informed decisions and guide their consumption using harm reduction approaches. To help accomplish this, the Lower-Risk Cannabis Use Guidelines (LRCUG) were developed, and contain a series of evidence-based guidelines and recommendations on reducing the risk of cannabis-related harms. The LRCUG contains twelve recommendations [18] and three precautionary statements, followed by evidence that supports these lower-risk behaviours. Each recommendation is given an evidence grade of limited, moderate, substantial, or conclusive. Examples of recommendations deemed moderate or substantial include consuming cannabis infrequently (e.g. once a week, only on weekends), and consuming low THC or high CBD/THC ratio cannabis.

Given that Canadian emerging adults have high consumption rates and are at a greater risk of experiencing cannabis-related harms, it is important to have a better understanding of this age group’s perceptions of higher-risk cannabis consumption behaviours. The LRCUG provides a strong evidence base in identifying specific higher-risk cannabis consumption behaviours and ways to mitigate potential harms. Previous vignette-based survey research has highlighted that emerging adults may not fully appreciate the risks of frequent cannabis consumption on mental health, cognitive health, or brain development [3, 4], nor the extent of the risks associated with driving under the influence of cannabis [19]. However, vignette-based survey research has yet to explore emerging adults’ perceptions of other higher-risk cannabis consumption behaviours identified in the LRCUG. As a result, we aim to address this gap within the current literature.

The current study builds on previous research on perceptions of potential harms by using an experimental vignette design to assess Canadian emerging adults’ perceived risk of five LRCUG behaviours that received an evidence grade of “moderate” or higher [18]. As such, the purpose of this study was to assess emerging adults’ perceptions of risk associated with: (1) frequency of consumption; (2) polysubstance use; (3) method of consumption; (4) family history of psychosis or a substance use disorder (SUD); and (5) potency of the cannabis product. Perceived risk was measured through the overall perceived risk of harm, and perceived risk to cognitive, physical, and mental health.

## Method

### Participants and recruitment

Individuals who self-identified as between the ages of 18 and 25 and currently living in Canada were eligible to participate. Recruitment was accomplished through social media sites (e.g. Facebook, Twitter, Instagram, etc.), email distribution lists, and distribution of QR codes at Memorial University of Newfoundland. Recruitment occurred between December 20, 2022, and January 29, 2023.

### Vignette development

Five sets of vignettes were developed based on the LRCUG recommendations and previous vignette research on perceptions of cannabis consumption risks [3, 19]. Each vignette depicted a 21-year-old character who consumed cannabis for non-medical purposes. Gender-neutral language was used to avoid gender-based biases [3, 20]. In each vignette, one independent variable was altered which included: (1) frequency of consumption (once a week, almost daily, or daily); (2) polysubstance use (cannabis and tobacco, cannabis and alcohol, or cannabis alone); (3) method of consumption (smoking, vaping, or ingesting edibles); (4) family history (a biological parent with a history of psychosis, a biological parent with a substance use disorder, or no family history of mental illness or substance-related concerns); and (5) potency (25% THC and 0.05% CBD, 5% THC and 10% CBD, or 45% THC and 0.01% CBD). The five sets of vignettes can be found in Additional file 1: Appendix A.

### Procedure

Individuals accessed the online survey through the Qualtrics platform. After reviewing the informed consent form, eligible emerging adults who consented to participate were directed to the survey. The survey consisted of five randomized vignettes, with one from each of the independent variables explored (frequency of consumption, polysubstance use, method of consumption, family history, and potency). Each vignette was followed by a set of risk perception questions. Participants were then asked a series of socio-demographic questions, including history of personal cannabis consumption.

### Measures

*Perceived Risk Questionnaire:* Perceptions of potential harms were assessed using four items that were adapted from the Perceived Risk Questionnaire which was used to measure perceptions of risk of frequent cannabis consumption among emerging adults [3]. Each item was measured using a five-point Likert scale. The first item measured overall perceived risk of the cannabis consumption behaviour (0 = Not at all dangerous, 4 = Extremely dangerous). The remaining items measured perceived impact on cognitive health, mental health, and physical health, and were reverse scored due to the order of scale anchors (4 = very negative impact, 0 = very positive impact). Across the five vignettes, Cronbach’s alpha ranged from α = 0.74 to α = 0.81.

*Cannabis Knowledge and History of Consumption***:** Participants were asked a series of socio-demographic questions to determine their: (1) perceptions of their knowledge of non-medical cannabis consumption (1 = Not at all, 10 = Extremely knowledgeable); (2) awareness of the LRCUG; (3) sources of cannabis knowledge (e.g. TV, social media, peers, family); and (4) history of cannabis consumption (yes or no). Participants who indicated that they had consumed cannabis within their lifetime were asked to report their age of first consumption and were directed to the Cannabis Use Disorder Identification Test-Revised (CUDIT-R).

*Cannabis Use Disorder Identification Test-Revised*: The CUDIT-R [21] is a screening tool that was used to assess participants’ frequency of cannabis consumption, associated consequences, and ability to stop using cannabis if desired. Scores range between 1 and 40, with scores greater than 8 suggesting “hazardous” cannabis consumption. Scores greater than 12 may be indicative of CUD [21]. Previous research has demonstrated that the CUDIT-R is reliable, and has been validated for use in emerging adult samples [22]. In the current sample, Cronbach’s alpha was 0.80.

### Data analysis

Analyses of frequency of missing data were conducted. Participants who did not meet eligibility criteria or respond to any vignette questions were removed from analyses. Participants who failed to respond to more than one item of the CUDIT-R were removed from analyses pertaining to this measure. Subsequently, data were assessed for outlier responses and outlier scores were winsorized [23].

Descriptive statistics were used to characterize the sample by demographic variables. Subsequently, a series of one-way ANOVAs and chi-squares were conducted to assess for group differences on demographic characteristics, including age, province or territory of residence, gender, ethnicity, student status, education level, previous cannabis consumption, age of first consumption, perceived cannabis knowledge, and CUDIT-R score. For vignettes with significant group differences between conditions, ANCOVAs were used to account for covariates.

A series of ANOVAs and ANCOVAs were conducted to assess emerging adults’ perceptions of the five higher-risk cannabis consumption behaviours on: (1) overall perceived risk; (2) impact on cognitive health; (3) impact on physical health; and (4) impact on mental health. Post hoc analyses were completed using Tukey’s HSD and Games-Howell tests. With a large sample size and, on average, over 200 participants per cell, the central limit theorem allows us to assume normal distribution with Likert scale ratings [23]. To account for multiple analyses, Bonferroni correction for multiple comparisons was employed, and a critical alpha of p < 0.0025 (0.05/20) was retained. Data analyses were completed using IBM SPSS version 29.0 [24].

## Results

**Fig. 1:**
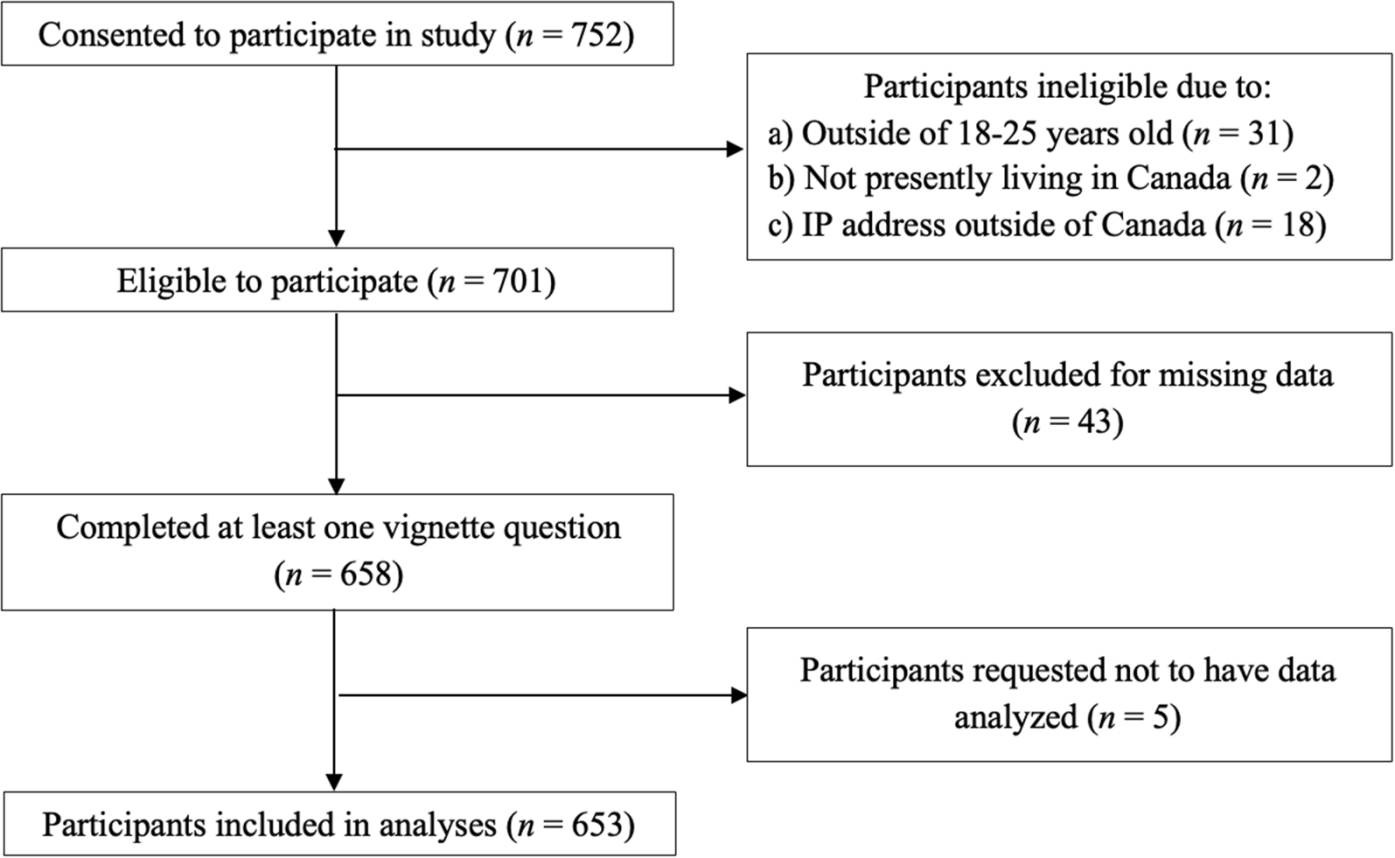
Analysis of missing data

From the 752 participants who responded, a total of 99 individuals were removed due to ineligibility, missing data (i.e. did not answer any vignette questions), or requested not to have their data analysed, leaving 653 who were included in the study (Fig. 1). Four participants began the CUDIT-R but failed to respond to more than one item and were therefore excluded from further analyses involving this measure. Further, due to detection of outlier data, 30 responses for the cognitive health measure and 24 responses for the physical health measure were winsorized across the five vignettes.

Canadian emerging adults (*n* = 653) between the ages of 18 and 25 (*M* = 20.85, *SD* = 2.01) participated in the current study, refer to Table 1. The majority of participants identified as female (66.6%), White (82.6%), students (94.3%) and had consumed cannabis in their lifetime (76.4%). Participants felt they were moderately knowledgeable about non-medical cannabis consumption (*M* = 5.53, *SD* = 2.23), and the two most common sources of cannabis knowledge were peers and social media. Additionally, most participants reported that they were not aware of the LRCUG (73.5%). Among those who reported previous cannabis consumption, the average age of first consumption was approximately 17 years of age. Participants who had consumed cannabis within the last six months completed the CUDIT-R (*n* = 349), with scores ranging from 1 to 32 (*M* = 8.59, *SD* = 6.08). Of those who completed the CUDIT-R, 30% (*n* = 104) reported consuming cannabis four or more times per week. Approximately 18% of participants who completed the CUDIT-R (*n* = 64) had scores that were classified as hazardous and an additional 26% (*n* = 94) had scores that suggested a possible CUD. As outlined in Table 2, analyses revealed significant group differences between the three conditions of the family history vignette based on participants’ gender, student status, and CUDIT-R score. Descriptive statistics (mean and standard deviation) for each vignette condition and domain of potential harm are detailed in Table 3. Sixteen ANOVAs and four ANCOVAs were conducted to assess the impact of various cannabis consumption behaviours on perceived risk of consumption. Results of these analyses are summarized in Table 4, and the post hoc analyses are summarized in Table 5.

**Table 1:** Participant characteristics

Demographic variable	*N* (Valid percent)
**Province**	
Alberta	9 (1.4)
British Columbia	6 (0.9)
Manitoba	4 (0.6)
New Brunswick	26 (4.0)
Newfoundland and Labrador	527 (80.7)
Nova Scotia	56 (8.6)
Ontario	12 (1.8)
Prince Edward Island	7 (1.1)
Quebec	1 (0.2)
Saskatchewan	2 (0.3)
Territories (Nunavut, Northwest Territories, Yukon)	3 (0.5)
**Gender**	
Woman	383 (66.6)
Man	151 (26.3)
Non-binary	15 (2.6)
Transgender	5 (0.9)
Self-identified (e.g. genderfluid)	3 (0.5)
Multiple gender identities	11 (1.9)
Preferred not to disclose	7 (1.2)
**Race/Ethnicity**	
White	475 (82.6)
Black	14 (2.4)
Asian (including South, East, and South-East Asian)	44 (7.7)
Hispanic or Latino	5 (0.9)
Middle Eastern	4 (0.7)
Self-identified ethnicity	10 (1.7)
Multiple ethnic identities	16 (2.8)
Preferred not to disclose	7 (1.2)
**Student status**	
Currently a student	543 (94.3)
Not currently a student	22 (5.7)
**Previous cannabis consumption**	
Yes	447 (76.4)
No	138 (23.6)
**Awareness of LRCUG**	
Yes	143 (24.8)
No	434 (75.2)
**Sources of cannabis knowledge**	
TV/movies	181 (27.7)
Academic journals	185 (28.3)
Peers	439 (67.2)
Podcasts	118 (18.1)
Government websites	200 (30.6)
Retailers	195 (29.9)
News outlets	144 (22.1)
School	258 (39.5)
Family	185 (28.3)
Social media	328 (50.2)
Other	22 (3.5)
**Demographic variable**	*M* ± *SD*
Age of first use	16.9 ± 2.33
Mean CUDIT-R score	8.59 ± 6.08
Perceived level of cannabis knowledge	5.53 ± 2.23
Age	20.85 ± 2.01
Years of education	15.62 ± 2.08

**Table 2:** Group differences on demographic variables

Demographic variable	Frequency of consumption		Polysubstance use		Family history		Method of consumption		Potency	
	χ^2^	*p*	χ^2^	*p*	χ^2^	*p*	χ^2^	*p*	χ^2^	*p*
Province	26.79	0.219	21.58	0.490	19.68	0.060	26.66	0.224	15.56	0.837
Gender	12.90	0.377	10.22	0.596	25.75	0.012*	12.85	0.380	11.23	0.509
Race/ethnicity	23.89	0.159	20.16	0.324	22.38	0.216	15.16	0.651	15.79	0.607
Student status	1.88	0.390	2.69	0.260	9.10	*0*.011*	0.92	0.632	1.14	0.566
Previous cannabis use	1.28	0.526	2.13	0.345	0.98	0.613	1.41	0.493	0.64	0.725

	*F*	*p*	*F*	*p*	*F*	*p*	*F*	*p*	*F*	*P*
Age	0.30	0.743	0.24	0.790	0.96	0.382	0.71	0.494	1.08	0.340
Education	0.14	0.867	2.75	0.065	0.49	0.613	0.54	0.580	0.76	0.470
Age of first consumption	0.08	0.928	0.97	0.381	0.23	0.793	0.35	0.705	0.04	0.957
CUDIT-R score	1.57	0.210	0.64	0.530	5.47	0.005**	0.64	0.528	1.86	0.157
Perceived level of cannabis knowledge	0.02	0.976	1.58	0.207	0.40	0.674	1.15	0.317	0.36	0.698

*p < 0.05, **p < 0.01, ***p < 0.001

**Table 3:** Level of perceived risk for cannabis consumption behaviours

Vignette	Overall harm M ± SD	Cognitive health M ± SD	Physical health M ± SD	Mental health M ± SD
**Frequency of consumption**				
Daily	2.61 ± 1.03	3.86 ± 0.68	4.01 ± 0.64	3.16 ± 1.22
Almost daily	2.92 ± 1.13	4.01 ± 0.70	4.18 ± 0.69	3.28 ± 1.25
Weekly	1.86 ± 1.00	3.59 ± 0.69	3.83 ± 0.66	2.89 ± 1.08
**Polysubstance use**				
Cannabis	1.71 ± 0.98	3.48 ± 0.64	3.67 ± 0.60	2.93 ± 0.95
Cannabis and tobacco	2.07 ± 1.01	3.56 ± 0.65	3.90 ± 0.63	3.07 ± 1.01
Cannabis and alcohol	1.92 ± 1.07	3.41 ± 0.67	3.67 ± 0.62	3.05 ± 0.94
**Family history**				
Psychosis	3.28 ± 1.09	4.10 ± 0.66	4.14 ± 0.70	3.99 ± 1.13
Substance use disorder	3.32 ± 1.00	4.11 ± 0.67	4.16 ± 0.64	3.94 ± 1.02
No family history	2.84 ± 1.12	3.98 ± 0.68	4.18 ± 0.65	3.67 ± 1.01
**Method of consumption**				
Edible	2.48 ± 1.17	3.98 ± 0.67	3.64 ± 0.70	3.44 ± 1.07
Vape	3.09 ± 1.15	3.99 ± 0.59	4.38 ± 0.62	3.59 ± 1.07
Smoke	3.17 ± 1.16	4.15 ± 0.66	4.38 ± 0.65	3.58 ± 1.14
*Potency*				
25% THC and 0.05% CBD	2.90 ± 1.14	3.98 ± 0.74	4.20 ± 0.70	3.54 ± 1.14
5% THC and 10% CBD	2.37 ± 1.06	3.71 ± 0.72	3.73 ± 0.75	3.19 ± 1.15
45% THC and 0.1% CBD	3.21 ± 1.08	4.18 ± 0.65	4.32 ± 0.67	3.80 ± 1.00

**Table 4:** ANOVA and ANCOVA results of perceived risk based on various factors of use

Vignette	One-Way ANOVAs			
	Measure	*F*	*p*	η^2^
Frequency of consumption	Overall harm	60.72	< 0.001*	0.16
	Cognitive health	21.33	< 0.001*	0.06
	Physical health	14.67	< 0.001*	0.05
	Mental health	6.78	0.001 *	0.02
Polysubstance use	Overall harm	7.11	< 0.001*	0.02
	Cognitive health	2.56	0.079	0.01
	Physical health	9.95	< 0.001*	0.03
	Mental health	1.24	0.292	0.04
Method of consumption	Overall harm	22.52	< 0.001*	0.07
	Cognitive health	4.32	0.014	0.01
	Physical health	84.79	< 0.001*	0.23
	Mental health	1.25	0.288	0.00
Potency	Overall harm	31.0	< 0.001*	0.09
	Cognitive health	23.7	< 0.001*	0.07
	Physical health	37.2	< 0.001*	0.12
	Mental health	16.2	< 0.001*	0.05

	One-Way ANCOVA			
	Measure/covariate	*F*	*p*	Partial η^2^
Family history	**Overall harm**	12.54	< 0.001*	0.07
	Gender	2.81	0.095	0.01
	Student status	0.25	0.619	0.00
	CUDIT-R score	1.35	0.246	0.00
	**Cognitive health**	0.82	0.443	0.01
	Gender	4.38	0.037	0.01
	Student status	0.04	0.850	0.00
	CUDIT-R score	0.27	0.601	0.00
	**Physical health**	0.51	0.602	0.00
	Gender	3.01	0.084	0.00
	Student status	0.92	0.340	0.00
	CUDIT-R score	0.29	0.590	0.00
	**Mental health**	4.91	0.008	0.03
	Gender	0.36	0.547	0.00
	Student status	2.21	0.138	0.01
	CUDIT-R score	0.35	0.557	0.00

Gender, student status, and CUDIT-R score were included as covariates for family history

*p ≤ 0.001, **p ≤ 0.0025, ***p ≤ 0.0001

**Table 5:** Post hoc analyses of effects of use factors on perceived risk of cannabis use

	Mean difference	*t*	*p*	*d*
**Frequency vignette predictor**				
*Overall risk of harm*				
Daily—almost daily	− 0.32	− 3.05	0.007	− 0.29
Daily—weekly	0.75	7.68	< 0.001*	0.74
Almost daily—weekly	1.07	10.56	< 0.001*	0.99
*Cognitive health*				
Daily—almost daily	− 0.15	− 2.29	0.059	− 0.22
Daily—weekly	0.28	4.14	< 0.001*	0.39
Almost daily—weekly	0.43	6.45	< 0.001*	0.60
*Physical health*				
Daily—almost daily	− 0.17	− 2.57	0.028	− 0.25
Daily—weekly	0.18	2.91	0.010	0.28
Almost daily—weekly	0.35	5.41	< 0.001*	0.52
*Mental health*				
Daily—almost daily	− 0.12	− 0.97	0.595	− 0.10
Daily—weekly	0.28	2.48	0.036	0.22
Almost daily—weekly	0.39	3.53	0.001*	0.33
**Polysubstance vignette predictor**				
*Overall risk of harm*				
Cannabis—cannabis and tobacco	− 0.36	− 0.36	< 0.001*	− 0.36
Cannabis—cannabis and alcohol	− 0.21	− 2.07	0.097	− 0.21
Cannabis and tobacco—cannabis and alcohol	0.15	1.45	0.314	0.14
*Physical health*				
Cannabis—cannabis and tobacco	− 0.23	− 3.896	< 0.001*	− 0.37
Cannabis—cannabis and alcohol	0.01	0.10	0.994	0.00
Cannabis and tobacco—cannabis and alcohol	0.24	3.82	< 0.001*	0.37
**Method of consumption vignette predictor**				
*Overall Risk of Harm*				
Edibles—vape	− 0.61	− 5.29	< 0.001*	− 0.53
Edibles—smoke	− 0.69	− 6.13	< 0.001*	− 0.59
Vape—smoke	− 0.08	− 0.64	0.801	− 0.07
*Physical health*				
Edibles—vape	− 0.74	− 11.30	< 0.001*	− 1.11
Edibles—smoke	− 0.74	− 11.54	< 0.001*	1.10
Vape—smoke	0.00	0.07	0.997	0
**Family history vignette**				
Overall Risk of Harm				
Psychosis—substance use disorder	0.13	0.99	0.583	− 0.04
Psychosis—no family history	0.65	4.75	< 0.001*	0.40
Substance use disorder—no family history	0.52	3.84	< 0.001*	0.45
**Potency vignette**				
*Overall risk of harm*				
25% THC and 0.05% CBD—5% THC and 10% CBD	0.53	4.79	< 0.001*	0.48
25% THC and 0.05% CBD—45% THC and 0.1% CBD	− 0.30	− 2.68	0.021	− 0.28
5% THC and 10% CBD—45% THC and 0.1% CBD	− 0.83	− 7.61	< 0.001*	− 0.78
*Cognitive health*				
25% THC and 0.05% CBD—5% THC and 10% CBD	0.27	3.86	< 0.001*	0.37
25% THC and 0.05% CBD—45% THC and 0.1% CBD	− 0.19	− 2.69	0.020	− 0.29
5% THC and 10% CBD—45% THC and 0.1% CBD	− 0.47	− 6.67	< 0.001*	− 0.69
*Physical health*				
25% THC and 0.05% CBD—5% THC and 10% CBD	0.47	6.53	< 0.001*	0.65
25% THC and 0.05% CBD—45% THC and 0.1% CBD	− 0.12	− 1.62	0.237	− 0.18
5% THC and 10% CBD—45% THC and 0.1% CBD	− 0.59	− 8.27	< 0.001*	− 0.83
*Mental health*				
25% THC and 0.05% CBD—5% THC and 10% CBD	0.35	3.02	0.008	0.31
25% THC and 0.05% CBD—45% THC and 0.1% CBD	− 0.26	− 2.37	0.048	− 0.24
5% THC and 10% CBD—45% THC and 0.1% CBD	− 0.61	− 5.70	< 0.001*	− 0.56

*p < 0.001, **p < 0.0025, ***p < 0.0001

*Frequency of Consumption:* There was a significant effect of frequency of consumption on each of the four one-way ANOVAs on overall risk, mental health, cognitive health, and physical health (refer to Table 4). Post hoc analyses (refer to Table 5) revealed that participants perceived: (1) daily consumption as having greater overall risks and a greater potential impact on cognitive health compared to weekly consumption; and (2) almost daily consumption as having greater overall risks and potential negative impacts on cognitive, physical, and mental health compared to weekly consumption.

*Polysubstance Use:* There was a significant effect of polysubstance use for one-way ANOVAs on overall risk and physical health (refer to Table 4). Post hoc analyses (see Table 5) suggested that participants perceived: (1) combining cannabis and tobacco to have greater overall risks and greater potential impacts on physical health compared to cannabis use alone; and (2) combining cannabis and tobacco as having greater potential impacts on physical health compared to combining cannabis and alcohol.

*Method of Consumption:* There was a significant effect of method of consumption for one-way ANOVAs on overall risk and physical health (refer to Table 4). Post hoc analyses (see Table 5) suggested that participants perceived smoking and vaping cannabis to have greater overall risks and greater potential impacts on physical health compared to ingesting edibles.

*Family History*: There was a significant effect of family history for one-way ANCOVAs on overall risk (refer to Table 4). Post hoc analyses (see Table 5) suggested that participants perceived a vignette character with a family history of psychosis or a family history of a SUD to have greater overall risks compared to a vignette character with no family history of mental illness or substance-related issues.

*Potency:* There was a significant effect of potency for one-way ANOVAs on overall risk, cognitive, physical, and mental health (see Table 4). Post hoc analyses (see Table 5) suggested that participants perceived: (1) 25% THC and 0.05% CBD cannabis as having greater overall risks and greater potential harms to cognitive and physical health compared to 5% THC and 10% CBD; and (2) 45% THC and 0.1% CBD as having greater overall risks and greater potential impacts on cognitive, physical, and mental health compared to 5% THC and 10% CBD.

## Discussion

Using the LRCUG to identify higher-risk cannabis consumption behaviours, the current study used an experimental vignette design to examine how Canadian emerging adults perceived different cannabis consumption behaviours that are associated with increased risk of harm. Our results suggested that emerging adults perceived greater risks of harm associated with higher frequency of cannabis consumption, combined consumption of cannabis and tobacco, and presence of a family history of either psychosis or SUD. Further, emerging adults viewed ingesting edibles and cannabis with 5% THC and 10% CBD as associated with the least risk of harm. While effect sizes for results were small, these findings help provide some insights into emerging adults’ cannabis knowledge and familiarity with the recommendations of the LRCUG. Further, findings may help to inform public health messaging and harm reduction substance education strategies among youth and emerging adults.

Encouragingly, the results of the current study suggested that emerging adults perceived almost daily consumption as having greater overall risk and potential impact on physical, cognitive, and mental health, compared to weekly consumption. However, while participants perceived daily consumption to have a greater overall risk and impact on cognitive health when compared to weekly consumption, they did not perceive any differences on the impacts to physical health and mental health. This finding is particularly concerning given that higher frequencies of consumption have been associated with an increased risk of psychosis [13], anxiety and depression [2], decreased grey matter volume resulting in decreased motivational, emotional, and affective processing [25], and increased risk of stroke [9] and bone fractures [10]. Interestingly, our finding is inconsistent with previous research suggesting that approximately 75% of emerging adults are aware of the impacts of daily or almost daily consumption on mental health [1]; ultimately highlighting the need to increase awareness among our sample of the risks of high-frequency (i.e., daily) consumption on mental and physical health.

With respect to polysubstance use, participants perceived consuming cannabis and tobacco together as having a greater overall risk and risks to physical health compared to cannabis consumption alone. This is encouraging as emerging adults seem to appreciate that using tobacco alongside cannabis can increase the risk of harm [26, 27]. However, participants did not perceive any difference in overall risk or risks to physical health between the combined consumption of cannabis and alcohol, compared to cannabis consumption alone. This lack of perceived difference is concerning as two-thirds of Canadians reported past-year simultaneous consumption of alcohol and cannabis [1], and combined consumption is associated with an increased risk of negative consequences [28, 29]. It is possible that our finding may be partially explained by the nature of the vignette, as the polysubstance use was described as taking place in a social setting and restricted to weekend consumption, which is often normalized in an emerging adult sample [30, 31]. Interestingly, participants did not perceive any differences in risk between cannabis and tobacco consumption compared to cannabis and alcohol consumption. Given that tobacco may have long-term effects, such as an increased risk of lung-related health issues [32], while alcohol may have been more readily associated with short-term effects, such as injury, it is possible that participants gave similar weight to these harms, resulting in a non-significant difference.

Notably, the only significant effect for the family history vignette was on overall risk, whereby participants perceived cannabis consumption with a family history of either psychosis or a SUD as having greater overall risk compared to cannabis consumption with no family history of mental illness or SUD. Initially, it was concerning to see no significant difference in perceptions of mental health risks between a character that had no family history of mental health or substance use concerns, compared to a family history of psychosis or SUD; however, across the three conditions, participants indicated higher ratings of risk to mental health compared to the scores of mental health risk in other vignettes. Given that the vignette conditions explicitly indicated “no family history of mental illness or substance-related issues”, “history of a substance use disorder”, or “history of a psychosis”, it seems likely that participants were primed to consider the mental health of the vignette character, ultimately impacting ratings of risk. Further, participants perceived similar levels of risk between using cannabis with a family history of psychosis or a family history of a SUD. This is a promising result as it suggests that participants viewed both as equally valid risks. In the past, cannabis was often perceived as non-addictive [33]; however, our finding suggests that emerging adults appreciate that cannabis can be addictive, which aligns with Statistics Canada data suggesting that 95% of Canadian emerging adults recognized that cannabis can be habit forming [1].

Consistent with previous research, emerging adults in our study perceived smoking and vaping cannabis to be associated with greater overall risks and impacts on physical health compared to ingesting edibles. This finding suggests that emerging adults were at least partially aware of the guideline to opt for edible cannabis instead of combustible cannabis. In our sample, there were no perceived differences across any domains of risk when comparing smoking and vaping cannabis. While this is inconsistent with some existing research [34, 35], it is consistent with the findings from a national survey, which suggests that smoking or vaping cannabis on a regular basis were perceived to be associated with the same level of risk, and consuming edibles on a regular basis was associated with decreased perceived risk of harm [1]. Relatedly, the impact of smoking on physical health has been well-documented in the literature, and over 80% of Canadian emerging adults recognized that cannabis smoke can be harmful [1].

In considering cannabis potency, emerging adults perceived consuming cannabis with 45% THC and 0.1% CBD as having greater overall risks and potential impacts on cognitive, physical, and mental health compared to consuming cannabis with 5% THC and 10% CBD. Similarly, emerging adults perceived consuming cannabis with 25% THC and 0.05% CBD as having greater overall risk and risks to cognitive and physical health compared to 5% THC and 10% CBD; however, they did not perceive any differences in risk to mental health. This finding suggests that emerging adults may be aware of some risks associated with a higher THC/CBD ratio or THC-dominant strains, but only partially aware of the association between increased potency and poorer mental health outcomes [10, 13, 16, 17, 36]. Further, emerging adults did not perceive a difference between consuming cannabis with 25% THC and 0.05% CBD and consuming cannabis with 45% THC and 0.1% CBD. This finding highlights a potential knowledge gap in our sample and aligns with previous survey data that indicates uncertainty about the impact of higher THC potency on impairment among emerging adults in Canada [1]. It is particularly concerning that emerging adults did not perceive a difference between the two more THC-dominant cannabis strains, and did not perceive greater risks to mental health with 25% THC and 0.05% CBD compared to 5% THC and 10% CBD, as high-potency cannabis has been associated with numerous mental health concerns, including increased risk of psychosis [10, 13], anxiety disorders [36], and severity of CUD [16, 17]. To maintain a consistent method of consumption across the potency vignettes (i.e., smoking), the highest potency cannabis was indicated to be hashish, as the potency of this product exceeds other forms of combustible cannabis. Hashish consumption is less common among emerging adults and participants may have been less aware of the harms associated with hashish [1]. Additionally, given that high-potency cannabis is classified as 20% THC and above, participants may not have been able to differentiate the risk of strains above this threshold [37]. Nevertheless, this finding remains troubling as: (1) cannabis strains are steadily increasing in THC content [38, 39]; (2) 60% of Canadian emerging adults report vaping high-potency cannabis oil in the past year; and (3) 35% of Canadian emerging adults report high THC and low CBD cannabis as their preferred cannabis composition [1]. Given the preference for and availability of high THC potency cannabis, it is concerning that our sample did not fully appreciate the risks associated with high-potency cannabis.

### Limitations and future directions

The current study has some important limitations. First, the sample was majority female, white, living in Newfoundland and Labrador, and currently enrolled as students, which limits the generalizability of our results to all emerging adults. Additionally, the study may have been biased towards cannabis consumers, given that our prevalence of cannabis consumption seems to be higher than the prevalence rates previously reported in Canadian emerging adults [1, 40]. However, the age of first consumption in our sample was similar to previous Canadian data [1]. Participants were recruited online, through social media, and through the distribution of QR codes on a university campus, which may have resulted in a sample that was of higher socioeconomic status and who were more informed and risk-averse than the general emerging adult population in Canada. As a result, the current study may provide an optimistic picture of the awareness of higher-risk cannabis consumption behaviours among the general population of emerging adults in Canada. The current study also did not collect information on potential confounding variables, including family or friends’ cannabis consumption or perceived benefits of cannabis consumption which may have influenced participants’ perceptions of risk. Future research should expand on emerging adult perceptions using a more diverse sample and collect additional demographic information to control for potential confounds.

While we found several statistically significant results, many of the effect sizes were relatively small, and the threshold for meaningful change resulting from risk perception is currently unknown. Consequently, while it appears that emerging adults were aware of many of the risks associated with cannabis consumption, these results may not represent substantial behaviour change. Future research should consider evaluating at which point changes in perceptions of risk may begin to influence cannabis consumption behaviour.

### Directions for improving cannabis health knowledge

Findings from the current study indicate that emerging adults in our sample seemed to be aware of some of the risks associated with cannabis consumption, but continued to under-appreciate the risks associated with some higher-risk cannabis consumption behaviours. As such, it appears that there is a continued need for more effective public health education to inform emerging adults’ perceptions of risk and promote awareness of harm reduction approaches. Previous research with youth in Newfoundland and Labrador has revealed that this group has a desire for empirically-informed content about cannabis to guide their decision-making. Specifically, youth in this sample desired information on: (1) harm reduction approaches; (2) cannabis properties; and (3) clear guidelines on the effects of cannabis [41].

The most common sources of cannabis knowledge in our sample were peers and social media. Previous research suggests that individuals who report these as their primary sources of knowledge are more likely to believe misinformation about cannabis [42]. Given that emerging adults often use social media to inform their substance use knowledge, it is critical to ensure they are provided with accurate information. Future efforts should be made to increase the availability and ease of access to evidence-based information on cannabis consumption, including access and awareness of the LRCUG, as ~ 25% of our sample had heard of the guidelines. Lastly, it is vital to implement up-to-date and empirically-informed education on cannabis in the education system to help inform youth at an early age and guide decision-making surrounding cannabis.

### Supplementary Information

**Additional file 1.** Vignettes developed for the current study.

## Data Availability

The raw data and materials are not openly available but can be made available upon reasonable request to the corresponding author.

## References

[1] Canadian Cannabis Survey (CCS) Detailed Tables [https://publications.gc.ca/collections/collection_2022/sc-hc/H21-312-2022-2-eng.pdf]

[2] Hall W, Leung J, Lynskey M (2020). The effects of cannabis use on the development of adolescents and young adults. Ann Rev Dev Psychol.

[3] Harris-Lane L, Winters E, Harris N (2021). Emerging adult perceptions of cannabis use based on age and sex of user. Emerg Adulthood.

[4] Harris-Lane LM, Drakes DH, Donnan JR, Rowe EC, Bishop LD, Harris N (2023). Emerging adult perceptions of cannabis consumption post-legalization: considering age and sex differences. J Adolesc Health.

[5] Leos-Toro C, Fong GT, Meyer SB, Hammond D (2020). Cannabis health knowledge and risk perceptions among Canadian youth and young adults. Harm Reduct J.

[6] Arain M, Haque M, Johal L, Mathur P, Nel W, Rais A, Sandhu R, Sharma S. Maturation of the adolescent brain. Neuropsychiatr Dis Treat 2013;449–461. 10.2147/NDT.S39776 PMC3621648 23579318

[7] Scott JC, Slomiak ST, Jones JD, Rosen AF, Moore TM, Gur RC (2018). Association of cannabis with cognitive functioning in adolescents and young adults: a systematic review and meta-analysis. JAMA Psychiat.

[8] Ghasemiesfe M, Ravi D, Vali M, Korenstein D, Arjomandi M, Frank J, Austin PC, Keyhani S (2018). Marijuana use, respiratory symptoms, and pulmonary function: a systematic review and meta-analysis. Ann Intern Med.

[9] Hemachandra D, McKetin R, Cherbuin N, Anstey KJ (2016). Heavy cannabis users at elevated risk of stroke: evidence from a general population survey. Aust N Z J Public Health.

[10] Sophocleous A, Robertson R, Ferreira NB, McKenzie J, Fraser WD, Ralston SH (2017). Heavy cannabis use is associated with low bone mineral density and an increased risk of fractures. Am J Med.

[11] Di Forti M, Marconi A, Carra E, Fraietta S, Trotta A, Bonomo M, Bianconi F, Gardner-Sood P, O'Connor J, Russo M (2015). Proportion of patients in south London with first-episode psychosis attributable to use of high potency cannabis: a case-control study. Lancet Psychiatry.

[12] Hosseini S, Oremus M (2019). The effect of age of initiation of cannabis use on psychosis, depression, and anxiety among youth under 25 years. Can J Psychiatry.

[13] Marconi A, Di Forti M, Lewis CM, Murray RM, Vassos E (2016). Meta-analysis of the association between the level of cannabis use and risk of psychosis. Schizophr Bull.

[14] Sideli L, Quigley H, La Cascia C, Murray RM (2020). Cannabis use and the risk for psychosis and affective disorders. J Dual Diagn.

[15] Hjorthøj C, Compton W, Starzer M, Nordholm D, Einstein E, Erlangsen A, Nordentoft M, Volkow ND, Han B. Association between cannabis use disorder and schizophrenia stronger in young males than in females. Psychol Med 2023;1–7. 10.1017/S0033291723000880 PMC10719679 37140715

[16] Arterberry BJ, Padovano HT, Foster KT, Zucker RA, Hicks BM (2019). Higher average potency across the United States is associated with progression to first cannabis use disorder symptom. Drug Alcohol Depend.

[17] Freeman TP, van der Pol P, Kuijpers W, Wisselink J, Das RK, Rigter S, van Laar M, Griffiths P, Swift W, Niesink R (2018). Changes in cannabis potency and first-time admissions to drug treatment: a 16-year study in the Netherlands. Psychol Med.

[18] Fischer B, Robinson T, Bullen C, Curran V, Jutras-Aswad D, Medina-Mora ME, Pacula RL, Rehm J, Room R, van den Brink W (2022). Lower-Risk Cannabis Use Guidelines (LRCUG) for reducing health harms from non-medical cannabis use: A comprehensive evidence and recommendations update. Int J Drug Policy.

[19] Pollard MA, Drakes DH, Harris N. Perceptions of the Risk and Social Acceptability of Driving Under the Influence of Cannabis. Int J Mental Health Addict 2022;1–18.

[20] Hemsing N, Greaves L (2020). Gender norms, roles and relations and cannabis-use patterns: a scoping review. Int J Environ Res Public Health.

[21] Adamson SJ, Kay-Lambkin FJ, Baker AL, Lewin TJ, Thornton L, Kelly BJ, Sellman JD (2010). An improved brief measure of cannabis misuse: the Cannabis Use Disorders Identification Test-Revised (CUDIT-R). Drug Alcohol Depend.

[22] Schultz NR, Bassett DT, Messina BG, Correia CJ (2019). Evaluation of the psychometric properties of the cannabis use disorders identification test-revised among college students. Addict Behav.

[23] Tabachnick BG, Fidell LS (2007). Using multivariate statistics.

[24] SPSS Statistics. 29 edition. Armonk, NY: IBM SPSS Statistics; 2022.

[25] Battistella G, Fornari E, Annoni J-M, Chtioui H, Dao K, Fabritius M, Favrat B, Mall J-F, Maeder P, Giroud C (2014). Long-term effects of cannabis on brain structure. Neuropsychopharmacology.

[26] Lemyre A, Poliakova N, Bélanger RE (2019). The relationship between tobacco and cannabis use: a review. Subst Use Misuse.

[27] Schlienz NJ, Lee DC (2018). Co-use of cannabis, tobacco, and alcohol during adolescence: policy and regulatory implications. Int Rev Psychiatry.

[28] Linden-Carmichael AN, Van Doren N, Masters LD, Lanza ST (2020). Simultaneous alcohol and marijuana use in daily life: implications for level of use, subjective intoxication, and positive and negative consequences. Psychol Addict Behav.

[29] Subbaraman MS, Kerr WC (2015). Simultaneous versus concurrent use of alcohol and cannabis in the National Alcohol Survey. Alcoholism Clin Exp Res.

[30] Terry-McElrath YM, Patrick ME (2018). Simultaneous alcohol and marijuana use among young adult drinkers: age-specific changes in prevalence from 1977 to 2016. Alcohol Clin Exp Res.

[31] Looby A, Prince MA, Villarosa-Hurlocker MC, Conner BT, Schepis TS, Bravo AJ (2021). Young adult use, dual use, and simultaneous use of alcohol and marijuana: An examination of differences across use status on marijuana use context, rates, and consequences. Psychol Addict Behav.

[32] Musk AW, De Klerk NH (2003). History of tobacco and health. Respirology.

[33] Wilkinson ST (2013). More reasons states should not legalize marijuana: medical and recreational marijuana: commentary and review of the literature. Mo Med.

[34] Nguyen N, Wong M, Delucchi K, Halpern-Felsher B (2022). Adolescents’ and young adults’ perceptions of risks and benefits differ by type of cannabis products. Addict Behav.

[35] Hammond D, Corsetti D, Goodman S, Iraniparast M, Danh Hong D, Burkhalter R. International Cannabis Policy Study—Canada 2021 Summary. In Cannabis Project; 2022.

[36] Wilson J, Freeman TP, Mackie CJ (2019). Effects of increasing cannabis potency on adolescent health. Lancet Child Adolescent Health.

[37] Cuttler C, LaFrance EM, Stueber A (2021). Acute effects of high-potency cannabis flower and cannabis concentrates on everyday life memory and decision making. Sci Rep.

[38] About cannabis [https://www.canada.ca/en/health-canada/services/drugs-medication/cannabis/about.html#]

[39] Mahamad S, Wadsworth E, Rynard V, Goodman S, Hammond D (2020). Availability, retail price and potency of legal and illegal cannabis in Canada after recreational cannabis legalisation. Drug Alcohol Rev.

[40] Mader J, Smith JM, Afzal AR, Szeto ACH, Winters KC (2019). Correlates of lifetime cannabis use and cannabis use severity in a Canadian university sample. Addict Behav.

[41] Bishop LD, Drakes DH, Donnan JR, Rowe EC, Najafizada M. Exploring Youths’ Cannabis health literacy post legalization: a qualitative study. J Adolescent Res 2022:07435584221118380. 10.1177/07435584221118380 PMC11637917 39678432

[42] Ishida JH, Zhang AJ, Steigerwald S, Cohen BE, Vali M, Keyhani S (2020). Sources of information and beliefs about the health effects of marijuana. J Gen Intern Med.

