# Supplementary material for: Emerging adult perceptions of higher-risk cannabis consumption behaviours
Source: Harm Reduct J. 2023 Sep 7;20:127. doi: 10.1186/s12954-023-00860-4 (PMC10483777; doi:10.1186/s12954-023-00860-4)
Supplement: Supplementary file 1 — Additional file 1. Vignettes developed for the current study. [file 12954_2023_860_MOESM1_ESM.docx]

Supplemental Appendix A

**Frequency of Consumption Vignettes**

1. A 21-year-old in your community consumes cannabis daily for non-medical (recreational) purposes.
2. A 21-year-old in your community consumes cannabis almost daily for non-medical (recreational) purposes.
3. A 21-year-old in your community consumes cannabis once a week for non-medical (recreational) purposes.

**Polysubstance Use Vignettes**

1. A 21-year-old in your community enjoys getting together with their friends on weekends. While together, they all consume non-medical (recreational) cannabis. Besides weekends with their friends, the 21-year-old does not consume cannabis.
2. A 21-year-old in your community enjoys getting together with their friends on weekends. While together, they all consume non-medical (recreational) cannabis and tobacco products. Besides weekends with their friends, the 21-year-old does not consume cannabis or tobacco products.
3. A 21-year-old in your community enjoys getting together with their friends on weekends. While together, they all consume non-medical (recreational) cannabis and drink alcohol. Besides weekends with their friends, the 21-year-old does not consume cannabis or drink alcohol.

**Family History Vignettes**

1. A 21-year-old in your community has one biological parent with a history of psychosis (e.g., a psychotic episode or schizophrenia). The 21-year-old consumes cannabis almost daily for non-medical (recreational) purposes.
2. A 21-year-old in your community has one biological parent with a history of a substance use disorder. The 21-year-old consumes cannabis almost daily for non-medical (recreational) purposes.
3. A 21-year-old in your community with no family history of mental illness or substance use related issues consumes cannabis almost daily for non-medical (recreational) purposes.

**Method of Consumption Vignettes**

1. A 21-year-old in your community ingests edible cannabis almost daily for non-medical (recreational) purposes.
2. A 21-year-old in your community vapes cannabis almost daily for non-medical (recreational) purposes.
3. A 21-year-old in your community smokes cannabis almost daily (e.g., joints, blunts, bongs) for non-medical (recreational) purposes.

**Potency of Cannabis Vignettes**

1. A 21-year-old in your community smokes dried cannabis flower with a 25% tetrahydrocannabinol (THC) content and a 0.05% cannabidiol (CBD) content almost daily for non-medical (recreational) purposes.
2. A 21-year-old in your community consumes dried cannabis flower with a 5% tetrahydrocannabinol (THC) content and a 10% cannabidiol (CBD) content almost daily for non-medical (recreational) purposes.
3. A 21-year-old in your community smokes cannabis extract (e.g. hashish) with a 45% tetrahydrocannabinol (THC) content and 0.1% cannabidiol (CBD) content almost daily for non-medical (recreational) purposes.
